# The H3 loop of antibodies shows unique structural characteristics

**DOI:** 10.1002/prot.25291

**Published:** 2017-04-06

**Authors:** Cristian Regep, Guy Georges, Jiye Shi, Bojana Popovic, Charlotte M. Deane

**Affiliations:** ^1^ Department of Statistics University of Oxford Oxford OX1 3LB United Kingdom; ^2^ Doctoral Training Centre, University of Oxford Oxford OX1 3QU United Kingdom; ^3^ Pharma Research and Early Development, Large Molecule Research, Roche Innovation Center Munich Penzberg 82377 Germany; ^4^ UCB Celltech Branch of UCB Pharma S.A. Slough SL1 3WE United Kingdom; ^5^ MedImmune Ltd., Department of Antibody Discovery and Protein Engineering Cambridge CB21 6GH United Kingdom

**Keywords:** antibodies, protein loop, CDR H3, structural diversity, loop modeling, Tyrosine, Glycine

## Abstract

The H3 loop in the Complementarity Determining Region of antibodies plays a key role in their ability to bind the diverse space of potential antigens. It is also exceptionally difficult to model computationally causing a significant hurdle for *in silico* development of antibody biotherapeutics. In this article, we show that most H3s have unique structural characteristics which may explain why they are so challenging to model. We found that over 75% of H3 loops do not have a sub‐Angstrom structural neighbor in the non‐antibody world. Also, in a comparison with a nonredundant set of all protein fragments over 30% of H3 loops have a unique structure, with the average for all of other loops being less than 3%. We further observed that this structural difference can be seen at the level of four residue fragments where H3 loops present numerous novel conformations, and also at the level of individual residues with Tyrosine and Glycine often found in energetically unfavorable conformations. Proteins 2017; 85:1311–1318. © 2017 Wiley Periodicals, Inc.

## INTRODUCTION

Antibodies are an essential part of the immune system. They are able to attain high specificity and affinity to almost any antigen. Over the last few decades development of therapeutic antibodies has grown rapidly and they now account for the majority of revenue in the sales of new bio‐therapeutics.^21^ The major drivers for the success of therapeutic antibodies is both their ability to bind to almost any target and their proven viability for protein design.^15^, ^18^, ^30^

A natural human antibody is a symmetric Y shape, each half of the symmetric unit has two chains: a heavy chain (H) and a light (L) chain. The majority of the affinity and specificity of antibodies is modulated by a set of binding loops called the Complementarity Determining Region (CDR) found on the variable domain of each of the two chains. There are six CDR loops, L1, L2, L3 on the light chain and H1, H2, and H3 on the heavy chain. Several definitions of the CDR loops have been proposed; they are based either on sequence variability, contacts with the antigen or structural variability (e.g., Refs. 1, 13, 19, 22, 25, 34). As the central theme of this work is structural variation we use the *Chothia* structural definition.^1^

Out of the six CDR loops, the H3 loop shows the greatest structural diversity and is located in the center of the binding site.^35^ It also gains the most mutations through affinity maturation^7^ and has on average the largest number of contacts with the antigen.^23^ It therefore plays a crucial role in antigen binding, making accurate modeling of H3 vital. However, the H3 loop is the only CDR for which computational methods consistently fail to produce sub‐angstrom models.^2^ Modeling for the other CDR loops is aided by the fact that the backbone structures can be clustered into a number of *canonical forms* (e.g., Refs. 6, 25, 26). Using just a few residues the canonical form and thus the structure of a CDR can be predicted relatively accurately. The H3 loop however does not show such canonical forms.

A number of theories for the difficulty in H3 modeling have been proposed. It is known that H3 loops sample a large number of conformations through the process of V(D)J recombination and somatic hyper‐mutation.^29^ It could be this larger diversity that prevents accurate modeling. A computational study has suggested that H3 loops are highly flexible, owing to their longer residue sequences and reduced number of stabilizing bonds.^3^ This could make modeling highly challenging. The length distribution of H3 is much broader than for other CDRs and the number of solved crystal structures could be too low to effectively allow for the clustering of shapes.^20^ In this article, we have analyzed H3 loop flexibility through a systematic study of the normalized temperature factor and show that H3 structures in the Protein Data Bank (PDB) are if anything less flexible than general protein loops.

Given that H3 is not more flexible than other loops we explored in detail what differentiates it. We compared the structures of the H3, the other five CDRs and 18 other loop sets from well populated superfamilies to a nonredundant set of structures from the PDB. We found that H3 contains by far the largest percentage of unique conformations (∼30%), on average 10 times more than the other loops. A kink in the C‐terminal end of CDR H3 has been previously hypothesized to be involved in H3 structural diversity.^28^, ^32^ Next, we analyzed the regions within the H3 loop which cause these differences. We found >1000 four residue fragments which adopt conformations not seen in any other structure. These fragments are consistently found in the area around the tip of the H3 loop and show a high propensity for Tyrosine and Glycine in unfavorable conformations. These results suggest that H3 loops present structural characteristics which are unique in the protein world and it is this uniqueness that allows antibodies to target the highly diverse space of antigen structures but also makes them difficult to model computationally.

## MATERIALS AND METHODS

### Datasets

#### Antibody CDRs

We took all the *F_v_* chains found in the SAbDab database^10^ on October 8, 2015 and removed those with resolution >3.0 Å. This resulted in 1779 structures with 4989 chains. From these chains the CDR loops were extracted according to the *Chothia* definition using the ANARCI numbering software.^9^ We discarded the CDR loops that have backbone atoms with a temperature factor higher than 80.0.

#### Loops from other superfamilies

Eighteen superfamilies were selected by randomly picking from those superfamilies that have >500 loops with unique sequences. We used the SCOP superfamily assignments^24^ and the Superfamily package^12^ to predict the superfamily for the chains in the PDB that do not have already have a manual assignment. Loops were then extracted from these chains as a region of more than three residues between two secondary structures as annotated by DSSP.^17^ The superfamilies and number of loops are detailed in Supporting Information Table S1.

#### Non‐IG like protein loops

For the comparison to general protein loops we used all the loops from every chain in the PDB that has a resolution better than 3.0 Å and is not IG‐like. We used DSSP^17^ as described above to define loops. Loops which have backbone atoms with a temperature factor higher than 80.0 were removed. We define a chain as being IG‐like if it is either in SabDab^10^ or contains in the description field terms related to MHCs or T‐Cell Receptors.

#### Bound loop definition

In some tests we split loops into bound and unbound. For antibodies a loop is considered to be bound if it is part of an antibody‐antigen complex as indicated by SabDab.^10^, ^22^ For non‐Ig proteins a loop is considered to be bound if any of its atoms are within 5.0 Å of any atom from a residues found on a different chain in the same PDB structure.

#### Nonredundant set of protein structures

A nonredundant set of protein structures was created by culling the chains in the PDB with resolution <3.0 Å at 90% sequence identity using PISCES.^31^ This resulted in 31,028 chains with an average number of 260 residues. From these chains we extracted all overlapping fragments between three and 30 residues.

### Temperature factor normalization and flexibility

The comparison of temperature factors between structures is difficult because the uncertainty of an atom position increases with a decrease in resolution (see Supporting Information Fig. S1). Hence, using the average temperature factor for comparing flexibility would be biased by resolution. We, therefore, normalized the value of each temperature factor to a Z‐score for the entire PDB file (the mean and the variance are calculated from all the temperature factors of the backbone atoms in the PDB structure) as suggested by Parthasarathy and Murthy.^27^ Using this method we observe that the normalized distribution does not vary with resolution.

Alternate conformations could potentially offer a more accurate picture of the flexibility of H3 loops, but there are very few structures that have backbone atoms for the H3 loop with multiple occupancies (one example is PDB structure with accession code 2VXU, chain H, residues 95 and 96), meaning it cannot be used at this time.

### Length matched sets

When comparing two sets of loops the result might be biased by the fact that their length distributions are different. To correct for this bias we generated length matched sets (LMS). If set B is compared to set A, and B has a different length distribution to A, a sample from B is randomly extracted without replacement such that at each residue length it matches the proportion of loops of that length in set A. For example if in set A 5% of loops have length 6, 3% length 9 and 2% length 12, then LMS(B) will be a sample of B which has 5% of loops at length 6, 3% at length 9 and 2% at length 12.

### Unique loop fragments

We define a fragment as a continuous chain of four amino acids. The set of fragments of a loop consists of all its overlapping four residue fragments (e.g., for a loop of length five there are two overlapping fragments of length four). Two fragments are considered to be structurally different if their Kabsch optimal superposition^16^ of the backbone atoms has an RMSD >1.0 Å. To identify if the H3 loop contains fragment conformations which are unique to the protein world we clustered all the fragments from non‐IG loops plus an anchor of two residues (both upstream and downstream). Over 12 million fragments were clustered into 64,830 unique shapes. Superposition, however, is not transitive and using a 1.0 Å cut‐off for clustering we might not capture some of the unique shapes. Therefore, when clustering the non‐IG fragments we chose a stricter uniqueness cut‐off of 0.5 Å. This is expected to result in a possible overestimation of the number of non‐IG shapes, reducing the possibility of generating false positives when classifying an H3 fragment as unique. We considered an H3 fragment to be unique when its closest structural neighbor from the cluster of non‐IG shapes is >1.0 Å RMSD.

### Dihedral angles

To define the expected dihedral angles in loops we took a nonredundant set of non‐IG loops and meshed their backbone atoms Φ−Ψ dihedral angle space into bins of 3.0 × 3.0 degrees. The frequency for each bin was computed and a 90% contour plot was generated. The algorithm for the contour plot used a greedy “highest‐frequency first” approach up to 90% of the density. If an angle falls out of the generated contour it is considered to be energetically unfavorable.

## RESULTS

### Flexibility

**Figure 1:**
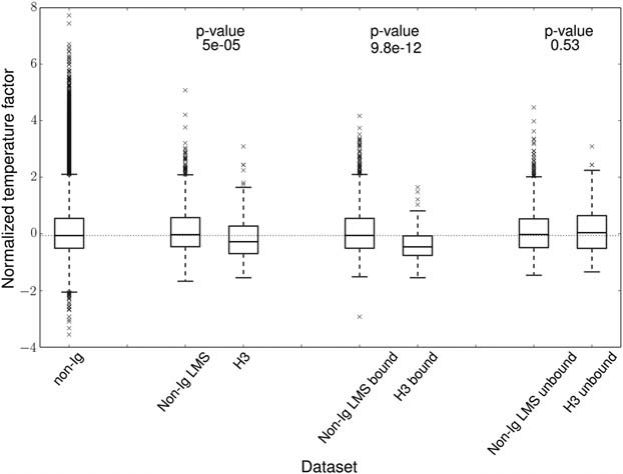
Flexibility comparison between H3 loops and non‐IG protein loops using the distribution of normalized temperature factors, one value per loop. For each of the H3, H3 bound, and H3 unbound datasets 10 length matches samples were generated from the non‐IG set and amassed to produce their associated LMS distribution: Non‐Immunoglobulin (non‐IG) LMS, non‐IG LMS bound, and non‐IG LMS unbound, respectively. Between each H3 loop set and its associated non‐IG LMS the *P*‐value from a two tailed Welch *t* test^33^ is reported.

We first tested using normalized temperature factors (see Materials and Methods) whether H3 loops are more flexible than other loops. Figure 1 shows how the distribution of normalized temperature factors of H3 loops compares to that of general protein loops (a LMS is also shown to correct for possible bias from the differences in length distribution). We find that the H3 loop does not show an increased flexibility. We also considered the potential bias induced by the fact that H3 loops are found in two states: bound and unbound. It has previously been suggested that loops involved in binding are less flexible. We, therefore, examined the bound and unbound H3 loops separately. We observe the expected increase of normalized temperature factor in the unbound H3 loops, however there is no significant difference to the behavior of unbound general protein loops (*P* value 0.53).

### Residue propensity and length distribution

We analyzed the length distribution and residue propensity distributions of all H3 loops. We compared these distributions to >200,000 loops from a nonredundant set of 25,361 PDB structures (see Materials and Methods and Supporting Information Fig. S2). H3 loops tend to be longer, peaking at length 10 as opposed to non‐IG loops which peak at length four. They also have a higher propensity for Tyrosine, Glycine, Aspartic Acid, and Phenylalanine. These differences have been previously reported in other studies (e.g., Refs. 35 and 5). However, if we carry out the same test for other CDRs (e.g., H2 or H1), H2 loops peak at length six and they have a higher propensity for Serine and Glycine than the general set. As all these sets are just subsets of the whole this result is perhaps not surprising but it suggests that it is not just length differences or particular amino acid preferences that are the reason for the difficulties in predicting H3 loops.

### Full loop structure

Given that H3 loops have a unique length and residue distribution we next looked at its structural divergence. For each of the H3 loops we computed the superposition and RMSD to every loop from all non‐IG chains in all crystal structures in the PDB with <3.0 Å resolution (2,281,826 loops). We did not cull the list of chains based on sequence because loops with the same sequence in different crystal structures can have different conformations (e.g., H3 loop in structure with PDB id 3v6f chain H and H3 loop in 3v6z chain C share the same sequence but have an RMSD of 2.69 Å). To represent how H3 loops and the other CDRs compare in terms of structural similarity to the rest of protein world, we plot distributions of minimum RMSD. For every loop in the query set we retained the value of the closest structural neighbor in all other proteins, excluding the query set. However, all CDRs apart from H3 adopt canonical forms (e.g., Refs. 6, 25, 26).

**Figure 2:**
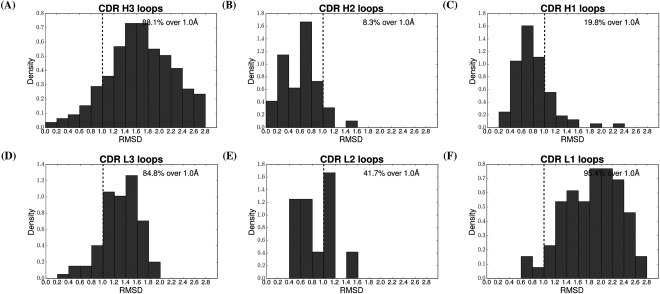
Structural similarity of CDR loops to non‐IG loops. For each CDR loop the closest structural neighbor in the rest of the protein world has been identified. The distribution of RMSD between the loops and their closest structural neighbor has been summarized as a histogram to show the structural similarity between the respective set of loops and general proteins. For each CDR the percentage of loops that have their closest structural neighbor at over 1.0 Å RMSD (the unique threshold) is reported. Shape duplicates have been removed in each data set. We define as shape duplicates sets of loops which have a superposition RMSD of less than 1.0 Å to another loop in the data set. For each set of such duplicates we retain only one loop. In the case of CDRs H1, H2, L1, L2, L3 this is approximately equivalent to retaining only one loop for each canonical class.

To check whether our results are biased by this we removed shape duplicates. Shape duplicates are sets of loops which have a superposition RMSD of <1.0 Å and for each set we retain only one loop. There are many definitions of canonical forms (these have been compared in several papers e.g., Ref. 26). We use a very simple 1.0 Å RMSD cut‐off as this is a standard definition of structural equivalence (e.g., Refs. 4, 8, 11, 14) and one which also provides a framework for including H3 in the analysis (which does not have canonical forms). Figure 2(A) shows this distribution for H3 loops is approximately normal, peaks around 1.5 Å, and 88% of the conformations are not found in the rest of the protein world. The other five panels in Figure 2(B–F) show the same data for the other CDRs. CDRs L1 and L3 also have most of their conformations >1.0 Å, while H1, H2, and L2 have most of their conformations under 1.0 Å. We also stratified this analysis by length to check for length bias (see Supporting Information Fig. S4), and this shows the same overall results. We also include in Supporting Information Figure S3 the results without shape duplicates removed, where for all but CDR L2 we observe similar overall results. The difference for CDR L2 is caused by the fact that there are only 7 unique shapes.

As L1 and L3 are known to take on canonical shapes it is likely if we allowed structures from the same superfamily (in this case the Ig fold) to be included we would expect L1 and L3 to have close structural neighbors whereas H3 may well still not. To show this we compared the CDRs to a nonredundant set of protein structures which include antibodies. This dataset consists of all overlapping fragments from 31,028 protein chains (includes secondary structure as well as loop—see Materials and Methods). Between 5.3 and 6.8 million fragments were compared to each loop (dependent on length). We found that H3 loops are structurally unique (have a closest structural neighbor with an RMSD >1.0 Å) at least 10 times more frequently than the other CDRs.

**Figure 3:**
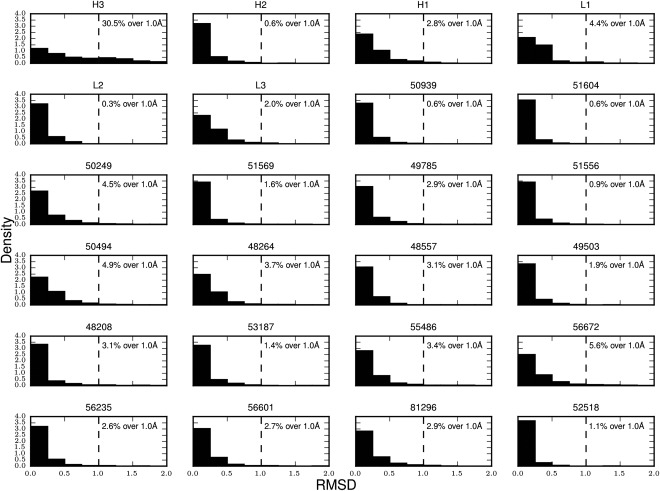
Structural similarity of the CDRs and 18 control loop sets to all fragments in a nonredundant set of PDB structures. For every loop in the sets of CDR loops and the 18 sets from other superfamilies a histogram of the RMSD of their closest structural neighbor from our nonredundant set of all protein structures is shown. The 18 control loop sets are from SCOP with the ID of the superfamily being provided as a title (details can be found in Table S1 of the Supporting Information). The percentage of loops with no close structural neighbor (> 1.0 Å RMSD) is given.

**Figure 4:**
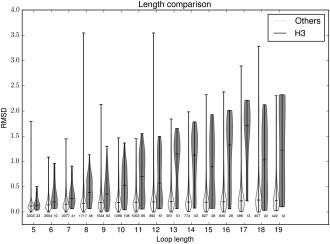
A violin plot comparing the difference in closest structural neighbor RMSD of H3 loops to the loops from the 18 control datasets at different lengths (see Fig. 3). At all lengths the H3 sets have on average a higher RMSD to their closest structural neighbors in the nonredundant set of protein structures.

To show that this diversity is not only unique for H3 in comparison to the other CDRs, but also in the general protein world we also selected 18 sets of loops from highly populated SCOP superfamilies (Supporting Information Table S1) and carried out the same test (Fig. 3). These loop sets also have only a small number of unique structures. The largest percentage of unique structures seen for anything other than H3 is 5.6%, and the average is approximately 3%. As H3 tends to be longer on average than other loops, we checked whether the observed structural difference was due to this length difference. Figure 4 shows that for all the lengths between five and 19 the closest structural neighbor to an H3 is on average further away than for other loops. We also checked whether our results might be affected by the fact that in each control set the loops are homologous. We performed an analysis where H3 is compared to five random samples of loops from all the superfamilies and we find that the same result holds (Supporting Information Fig. S5).

The challenge of modeling H3 appears to arise from its structural novelty. These results show that even if a perfect scoring system existed such that we could always select the closest structural neighbor as a prediction we would fail to achieve sub‐Angstrom accuracy at least 75% of the time if we used only non‐IG loops as the prediction library, and at least 30% of the time otherwise.

### Unique fragment conformations

**Figure 5:**
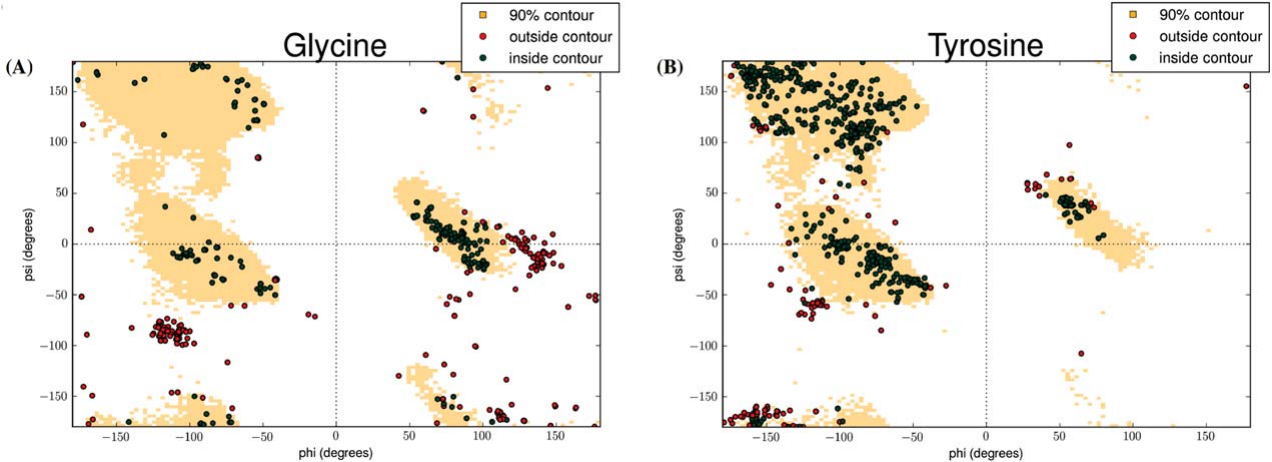
Ramachandran plots of Glycine (A) and Tyrosine (B) residues generated from a nonredundant set of protein loops. Each dot on the plot is a residue from a unique H3 fragment. A red dot indicates a residue with a conformation which is outside the 90% contour and, therefore, considered potentially energetically unfavorable. A green dot indicates a residue which is inside the 90% contour.

Next we tested whether the entire H3 or only segments of the loop are structurally unique. We extracted all the four residue overlapping fragments from every H3 loop and compared it to the set of 64,830 structurally unique four residue segments found in the rest of the PDB (see Materials and Methods). We identified a list of >1000 fragments that are unique to H3, with >30% of H3 loops containing at least one unique fragment. Supporting Information Figure S6 shows the characteristics of these fragments. The fragments tend to occur close to the tip of the H3 loop. We define the tip as the residue in the loop that contains the *C_α_* at the greatest distance from the *C_α_* of the residues at the start and end of H3. To identify whether these unique H3 fragments have a sequence preference we calculated their amino acid propensities. We observed that the unique fragments have a high propensity for Tyrosine and Glycine, even when compared to the rest of the H3 fragments [Supporting Information Fig. S6(A)]. Tyrosine and Glycine are known to have a high propensity throughout H3 [Supporting Information Fig. S2(B)], but our result suggests that they are even more concentrated within the unique fragments. Examining these residues we found that the unique fragments contain large numbers of Tyrosine and Glycine adopting energetically unfavorable ϕ−ψ angle combinations (Fig. 5). These fragments are not more flexible then the other H3 fragments when comparing normalized temperature factors (Supporting Information Fig. S7). It appears that the unique fragments and thus unique H3 conformations may arise from these residues and dihedral patterns.

## DISCUSSION

The H3 CDR loop in antibodies is often the most important loop for antigen binding. Through the process, which is unique to antibodies, of V(D)J recombination and somatic hypermutation the CDR loops (including the H3) are refined to achieve high affinity and specificity to target antigens. To be able to modulate binding to a very large palette of potential antigens the H3 is known to have very high structural variability. It has been previously suggested that the source of its structural variability is an increased flexibility because of its longer length and lack of stabilizing bonds. However, the same study suggested that affinity matured antibodies present rigid backbone conformations. What we observe is that the antibodies present in the PDB do not show an increased flexibility when compared to general protein loops. This could be because most crystallized antibodies are matured high affinity binders. Nevertheless, high flexibility is not present and can not explain the difficulty in modeling the H3 loops of the structures in the PDB.

What we did identify is that H3 loops are distinctive in their structural characteristics and diversity from other loops. Thirty percent of H3 loops are unique compared to a nonredundant set of the PDB structures, on average 10 times more than our control datasets. Also, 75% of these H3 loops do not have a sub‐Angstrom structural neighbor in non‐IG proteins. This result is mirrored by the fact that some of the best predictions in the Antibody Modeling Assessment^2^ relied on physics‐based approaches. To try and understand the origin of these unique H3 structures we examined all four residue fragments from H3s and found >1000 unique four residue fragments. These fragments have conformations which are not seen in the rest of the PDB. A high proportion of these fragments are found in close proximity to the tip of the H3 loop. We also observed that these fragments have increased levels of Tyrosine and Glycine compared to other H3 fragments which already have high levels of these amino acids. The uniqueness is further cemented by the fact that these residues are seen to adopt energetically unfavorable dihedral angles, which could be the reason for the structural diversity we observe. These results are a strong indication that the use of fragments of known structure from non‐IG proteins will not be effective in attempts to model the H3 loop to sub‐Angstrom accuracy. There is, therefore, a necessity to develop methods which focus specifically on the characteristics of these unique loops.

## Supporting information

Supporting Information
