# Supplementary material for: The H3 loop of antibodies shows unique structural characteristics
Source: Proteins. 2017 Apr 6;85(7):1311–8. doi: 10.1002/prot.25291 (PMC5535007; doi:10.1002/prot.25291)
Supplement: Supplementary file 1 — Supporting Information [file PROT-85-1311-s001.pdf]

SUPPLEMENTARY INFORMATION

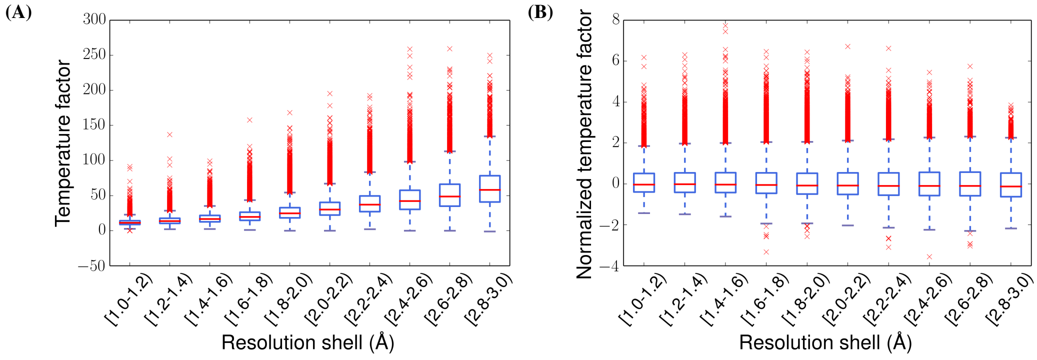

Figure S1: Related to Figure 1. Variation of temperature factor (A) and normalized temperature factor (B) with increase of resolution in a non-redundant set of protein loops. The temperature factor is seen to increase with the resolution of the structure while the normalized temperature factor averages around zero for all resolution shells.

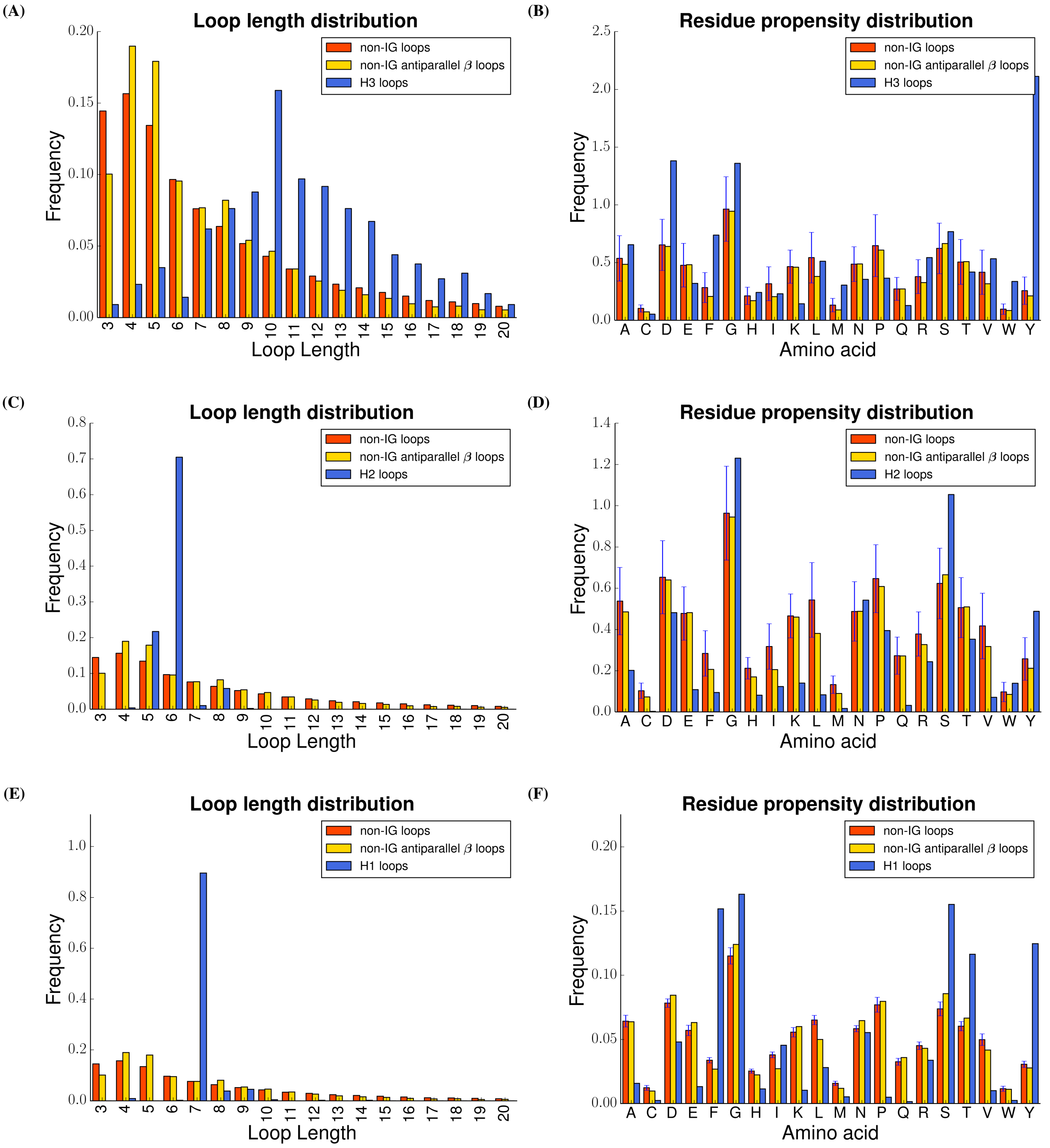

Figure S2: Length distributions and residue propensity of H3 (A,B), H2 (C,D) and H1 (E,F) compared to non-IG loops (see Materials and Methods). For residue propensity distributions the error bars are obtained by generating length matched samples.

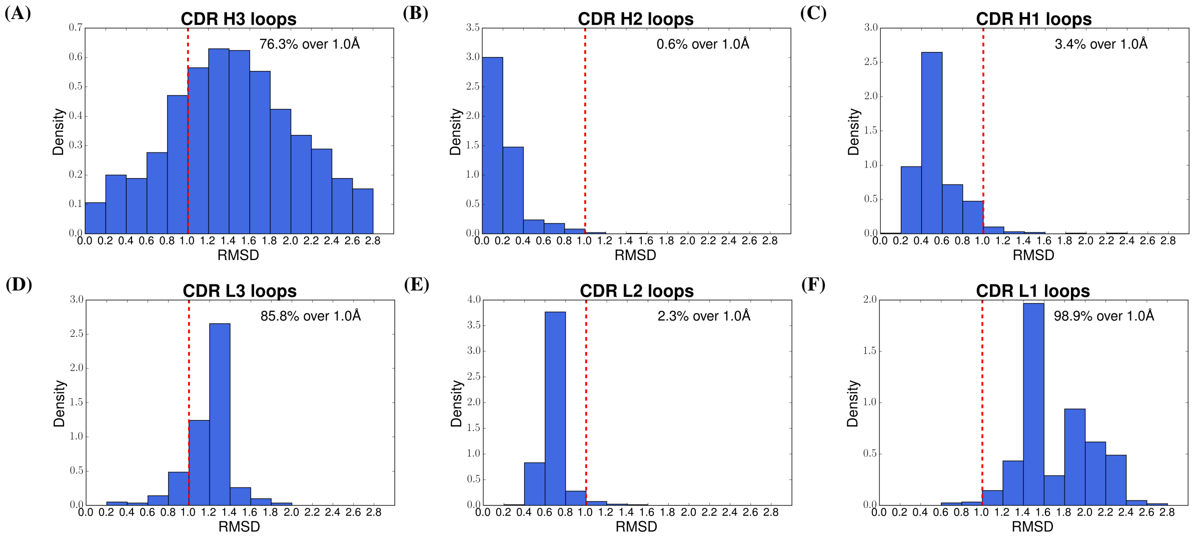

Figure S3: Results of the same analysis performed for Figure 2 without removing shape duplicates.

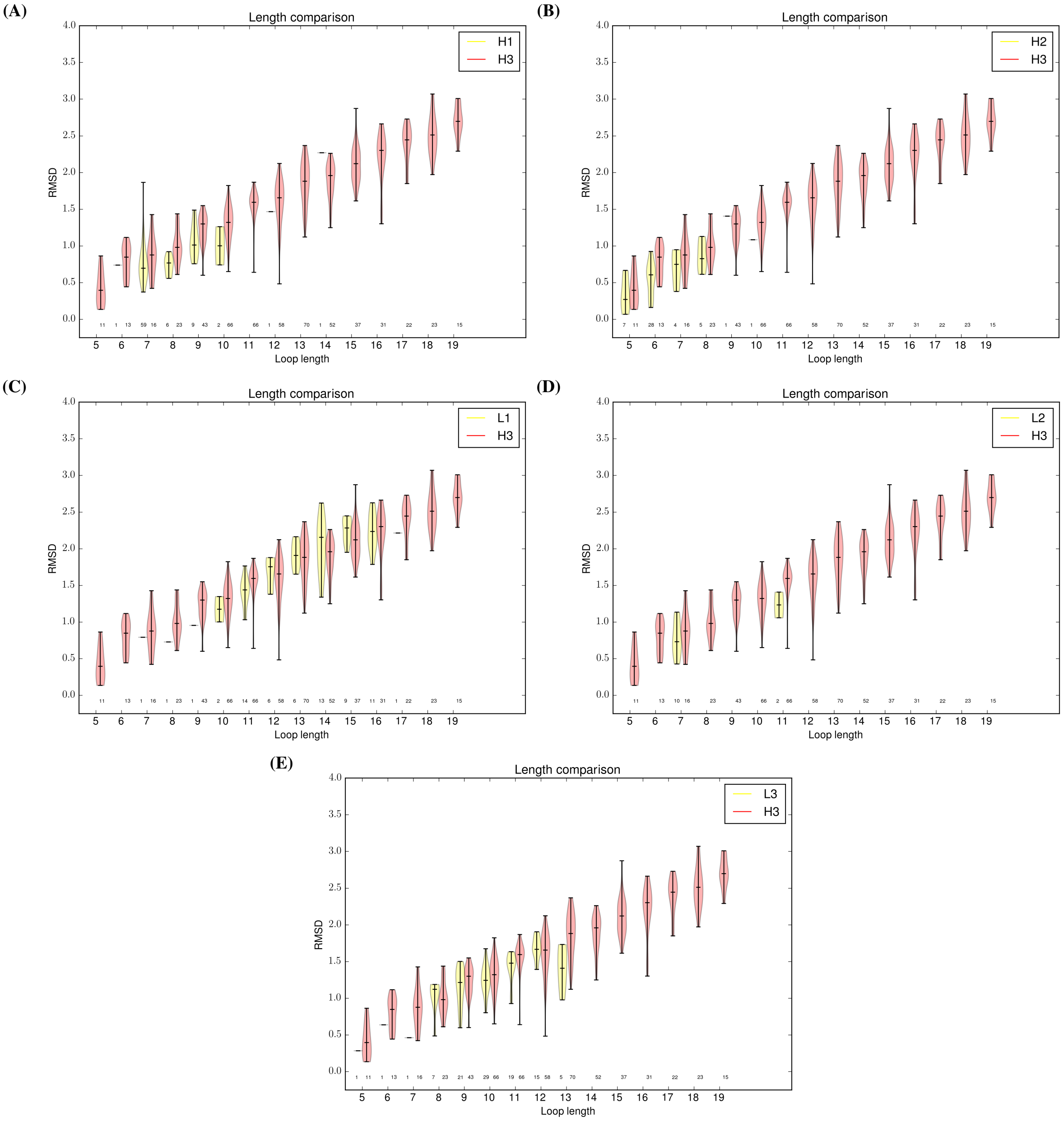

Figure S4: Violin plots showing the results from figure S3 split by loop length.

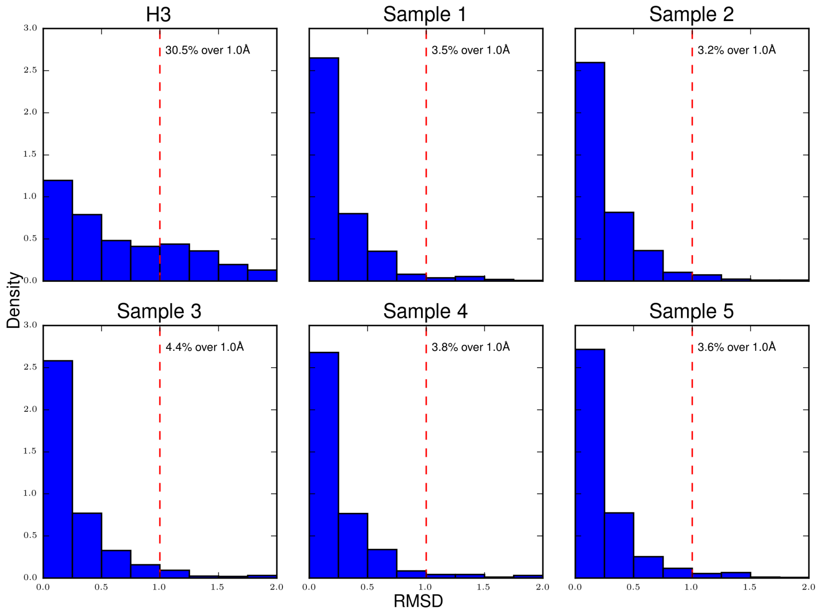

Figure S5: The same analysis performed in Figure 3, but with the Sample data sets being generated randomly from across all the 18 control data sets in table S1 and matching the length distribution of the H3.

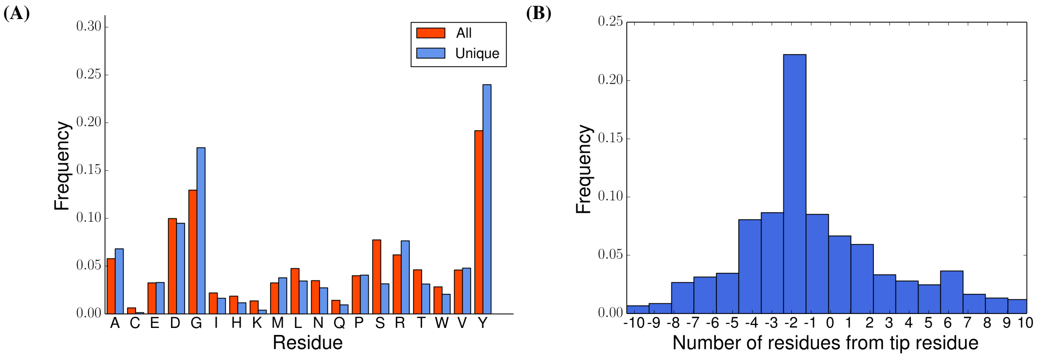

Figure S6: (A) Residue propensity distribution of the four residue H3 fragments in unique conformations (blue) and all of the H3 (red). (B) Distribution of the distance of the first C_α in a unique fragment from the tip of the loop.

Table S1: Relating to figure 3. Description of the superfamilies used as controls.

SCOP ID	Superfamily Name	Fold	Number of loop structures
50939	Sialidases	All β	979
51604	Enolase C-terminal domain-like	α/β	1976
50249	Nucleic acid-binding proteins	All β	896
51569	Aldolase	α/β	2829
49785	Galactose-binding domain-like	All β	1125
51556	Metallo-dependent hydrolases	α/β	2323
50494	Trypsin-like serine proteases	All β	2060
48264	Cytochrome P450	All α	1746
48557	L-aspartase-like	All α	850
49503	Cupredoxins	All β	1303
48208	Six-hairpin glycosidases	All α	1044
53187	Zn-dependent exopeptidases	α/β	1475
55486	Metalloproteases ("zincins"), catalytic domain	$\alpha+\beta$	1380
56672	DNA/RNA polymerases	Multi-domain	1971
56235	N-terminal nucleophile aminohydrolases	$\alpha+\beta$	1539
56601	beta-lactamase/transpeptidase-like	Multi-domain	1809
81296	E set domains	All β	1022
52518	Thiamin diphosphate-binding fold (THDP-binding)	α/β	1396

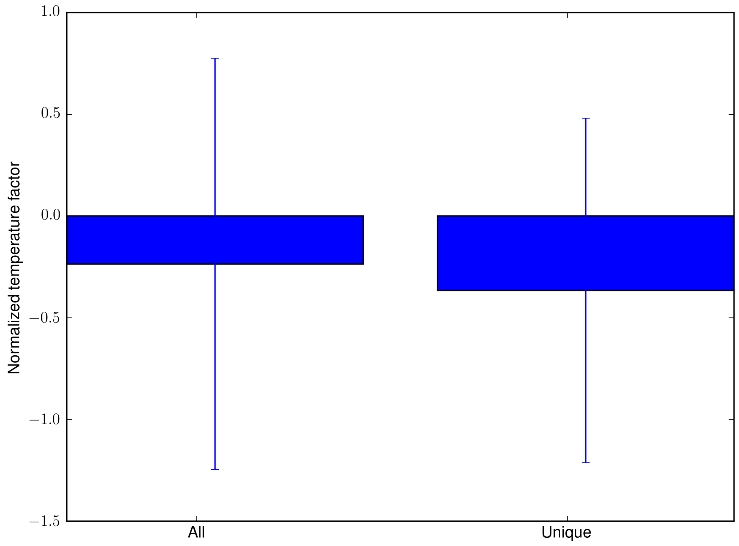

Figure S7: Relating to figure 5. Normalized temperature factor for unique fragments in comparison with all the H3 fragments
